# Nuclear AIM2‐Like Receptors Drive Genotoxic Tissue Injury by Inhibiting DNA Repair

**DOI:** 10.1002/advs.202102534

**Published:** 2021-10-18

**Authors:** Hui Jiang, Patrycja Swacha, Nelson O. Gekara

**Affiliations:** ^1^ Department of Molecular Biosciences The Wenner‐Gren Institute Stockholm University Stockholm 106 91 Sweden

**Keywords:** AIM2‐like receptors, cancer, chemotherapy, chromatin compaction, DNA repair, inflammasome, radiotherapy

## Abstract

Radiation is an essential preparative procedure for bone marrow (BM) transplantation and cancer treatment. The therapeutic efficacy of radiation and associated toxicity varies from patient to patient, making it difficult to prescribe an optimal patient‐specific irradiation dose. The molecular determinants of radiation response remain unclear. AIM2‐like receptors (ALRs) are key players in innate immunity and determine the course of infections, inflammatory diseases, senescence, and cancer. Here it is reported that mice lacking ALRs are resistant to irradiation‐induced BM injury. It is shown that nuclear ALRs are inhibitors of DNA repair, thereby accelerate genome destabilization, micronuclei generation, and cell death, and that this novel function is uncoupled from their role in innate immunity. Mechanistically, ALRs bind to and interfere with chromatin decompaction vital for DNA repair. The finding uncovers ALRs as targets for new interventions against genotoxic tissue injury and as possible biomarkers for predicting the outcome of radio/chemotherapy.

## Graphical abstract

AIM2‐like receptors (ALRs) are key players in innate immunity. It is shown that nuclear ALRs are regulators of chromatin structure and repair, and thereby accelerate genome destabilization and cell death, independently of their role in innate immunity. The finding uncovers ALRs as targets for new interventions against genotoxic tissue injury and as possible biomarkers for predicting the outcome of radio/chemotherapy.

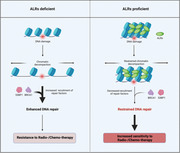

## 1 Introduction

Bone marrow (BM) injury is a key consequence of standard radiotherapy and chemotherapy for BM transplantation and cancer treatment. The sensitivity to irradiation or chemotherapy varies from individual to individual, mostly due to inherent genetic or epigenetic differences.^[^ ^1^ ^]^ Hence, the fixed‐dose prescribed for the majority is limited because of the severe toxicity in a minority of individuals.^[^ ^1^ ^]^ Understanding the genetic determinants of sensitivity to radiation or chemotherapy is vital for optimal individualized treatments and for the development of new avenues to prevent or treat genotoxic tissue injury.

AIM2‐like receptors (ALRs) are a large family of structurally related proteins generally thought to act as intracellular DNA sensors which alert the innate immune system to the presence of DNA in the cytosol of infected or stressed cells.^[^ ^2^ ^]^ The ALR family constitutes four members in humans (AIM2, IFI16, IFIX (PYHIN1), and MNDA) and thirteen in mice (including Aim2, p202, p203, p204, p205, p207, pyhin1).^[^ ^2a,d^ ^]^ Of these, the best characterized is AIM2 (absent in melanoma 2), the intracellular DNA sensor which signals via the adaptor protein ASC (Apoptosis‐associated speck‐like protein containing a CARD), to activate the inflammasome, a key arm of the innate immune system.^[^ ^2b,c^ ^]^ However, whether the other ALRs also function as bona fide innate immune DNA receptors are still contentious, raising the question of whether these structurally related proteins might have a common function outside innate immunity.

In this study, we demonstrate that ALRs have a distinct and common function outside the immune system: regulation of chromatin structure and repair. We show that mice and cells lacking ALRs repair DNA breaks more efficiently and hence are more resistant to the genotoxic effects of irradiation and chemotherapy.

## 2 Results

### 2.1 ALRs Drive Radiation‐Induced BM Injury Independently of Inflammasome

**Figure 1:**
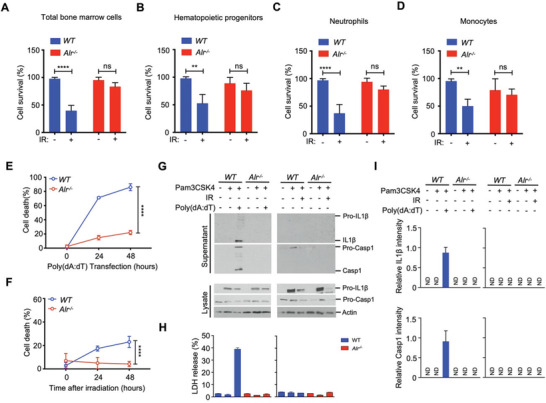
ALRs drive radiation‐induced cell death and BM injury. A–D) Survival of BM cells in WT (*n* = 8) and *Alr^–/–^* (*n* = 8) mice 10 h post‐*γ*‐irradiation (9 Gy). E) Cell death in WT and *Alr^–/‐^* BMDMos transfected with poly(dA:dT) (*n* = 3, biological repeats). F) Cell death in WT and *Alr^–/‐^* BMDMos following *γ*‐irradiation (9 Gy) (*n* = 3, biological repeats). G) Representative immunoblots of Caspase‐1 and IL‐1*β* processing in WT and *Alr^–/‐^* BMDMos following poly(dA:dT) transfection or *γ*‐irradiation (9 Gy). H) LDH release by WT and *Alr^–/–^* BMDMos following poly(dA:dT) transfection or *γ*‐irradiation (9 Gy). I) Corresponding quantification of immunoblots in (G) (*n* = 3, biological repeat). ND: non detectable. Data in (A–D) are presented as mean ± sd. Statistical significance is assessed using one‐way ANOVA followed by Tukey's multiple comparisons test. Statistical significance in (E,F) is assessed using two‐way ANOVA test. ns: not significant, **P < 0.01, ****P < 0.0001. See also Figure S1, Supporting Information.

**Figure 2:**
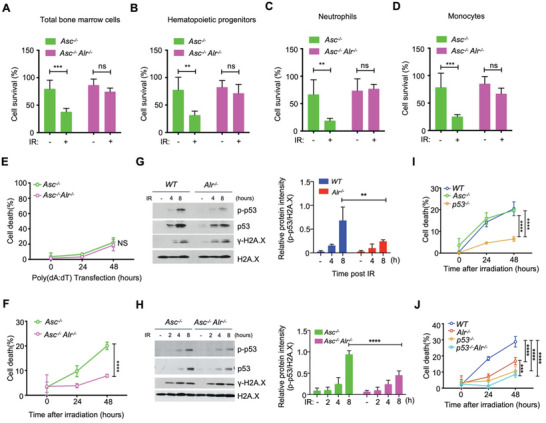
ALRs drive radiation‐induced cell death via p53 and not inflammasome activation. A–D) Survival of BM cells in *Asc^–/–^* (*n* = 10) and *Asc^–/–^Alr^–/–^* (*n* = 10) mice 10 h post‐*γ*‐irradiation (9 Gy). E) Cell death in *Asc^–/–^* and *Asc^–/–^Alr^–/–^* BMDMos transfected with poly(dA:dT) (*n* = 3, biological repeat). F) Cell death in *Asc^–/–^* and *Asc^–/–^Alr^–/–^* BMDMos following *γ*‐irradiation (*n* = 3, biological repeat). G) Immunoblots (left) and corresponding quantification (right) of p53 and H2A.X phosphorylation in WT and *Alr^–/–^* BMDMos following *γ*‐irradiation (*n* = 3, biological repeat). H) Immunoblots (left) and corresponding quantification (right) of p53 and H2A.X phosphorylation in *Asc^–/–^* and *Asc^–/–^Alr^–/–^* BMDMos following *γ*‐irradiation (9 Gy) (*n* = 3, biological repeat). I) Cell death in WT, *Asc^–/–^*, and *p53* ^–/–^ BMDMos following *γ*‐irradiation (9 Gy) (*n* = 3, biological repeat). J) Cell death in WT, *Alr^–/–^*, *p53* ^–/–^, and *p53* ^–/–^ *Alr^–/–^* BMDMos following *γ*‐irradiation (9 Gy) (*n* = 3, biological repeat). Data in A–D) are presented as mean ± sd. Statistical significance is assessed using one‐way ANOVA followed by Tukey's multiple comparisons test. Statistical significance in E–J) is assessed using two‐way ANOVA test. ns: not significant, **P < 0.01, ***P < 0.001, ****P < 0.0001.

While investigating the molecular determinants of radio‐sensitivity, we found that mice lacking ALRs (*Alr^–/–^*)^[^ ^2d^ ^]^ were highly resistant to irradiation‐induced BM injury (**Figure** **1**A–D). AIM2 inflammasome has previously been implicated in irradiation‐induced cell death.^[^ ^2e,f^ ^]^ Thus, to address whether ALR‐driven genotoxic cell death was inflammasome mediated, first, we assessed inflammasome activation in BM‐derived monocytes (BMDMos) following *γ*‐irradiation or poly(dA:dT) transfection (a canonical AIM2 agonist). While both poly(dA:dT) or *γ*‐irradiation (9 or 20 Gy) could induce cell death in WT and not *Alr^–/–^* BMDMos, only poly(dA:dT) was found to evoke detectable inflammasome response (Figure 1E–I and Figure S1A–F, Supporting Information). This suggested that ALR‐driven genotoxic cell death was likely independent of inflammasome activation. To interrogate this idea conclusively, we crossed the *Alr^–/–^* mice with the *Asc^–/–^* mice lacking the adaptors ASC. We found *Asc^–/–^Alr^–/–^* mice to be highly resistant to irradiation‐induced BM injury in contrast to *Asc^–/–^* which responded comparably to WT mice (**Figure** **2**A–D and Figure 1A–D). Similarly, irradiation evoked cell death in *Asc^–/–^* BMDMos, but hardly in *Asc^–/–^Alr^–/–^* BMDMos, in contrast to poly(dA:dT) transfection which hardly induced cell death in *Asc^–/–^* or *Asc^–/–^Alr^–/–^* BMDMos (Figure 2E,F). Thus, although inflammasome has previously been implicated in irradiation‐induced injury of some tissues^[^ ^2^, ^3^ ^]^, the present data demonstrate that the acute injury to the BM cells following irradiation is largely inflammasome‐independent.

### 2.2 ALRs Drive Genotoxic Cell Death Mainly via p53

Since p53 is a key effector of the DNA damage‐induced cell death,^[^ ^4^ ^]^ next we assessed whether ALRs had an impact on p53 activation. Compared to WT or *Asc^–/‐^* BMDMos, irradiation‐induced p53 activation was lower in *Alr^–/–^* or *Asc^–/–^Alr^–/–^* BMDMos (Figure 2G,H). In contrast to the *Asc^–/–^* BMDMos which responded comparably to WT BMDMos, *p53^–/–^* BMDMos were highly resistant to irradiation‐induced cell death (Figure 2I). To conclusively assess whether ALR‐driven genotoxic cell death was p53‐mediated, we crossed *Alr^–/–^* with *p53^–/–^* mice and compared the sensitivity of WT, *Alr^–/–^*, *p53^–/–^*, and *Alr^–/–^p53^–/‐^* BMDMos to irradiation or doxorubicin, a genotoxic anti‐cancer agent. Ablation of p53 decreased ALR‐driven genotoxic cell death in BMDMos (Figure 2J, Figure S2A, Supporting Information). ALRs are often lost in cancer cells.^[^ ^5^ ^]^ Thus, to extend our findings, we asked if exogenous expression of ALRs in the HCT116 cells which lack many components of the innate immune system including ALRs and inflammasomes could promote genotoxic cell death in these cancer cells. Expression of ALRs promoted doxorubicin – induced killing of HCT116 cells, indicating that ALRs do sensitize cells to DNA damage‐based anti‐cancer therapy (Figure S2B, Supporting Information). Further, analysis of publicly available database (kmplot.com/analysis) using the Kaplan–Meier Plotter^[^ ^6^ ^]^ revealed that higher expression of ALRs does correlate with better patient survival following chemotherapy (Figure S2C–F, Supporting Information). Together, these data demonstrate that ALRs are important for the induction of genotoxic cell death in primary as well as cancer cells and that this effect is mainly via p53 and not via inflammasome activation.

### 2.3 ALRs Promote Micronuclei Generation

**Figure 3:**
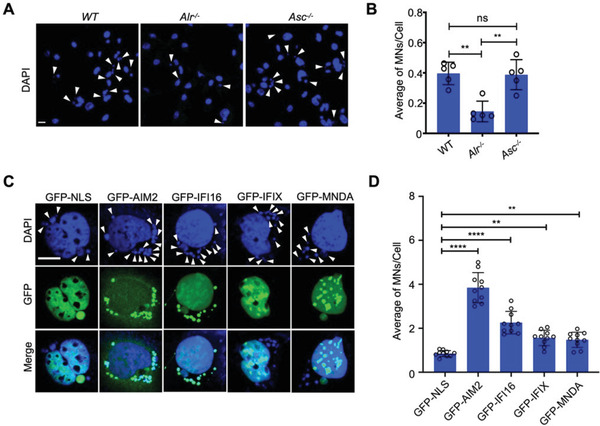
ALRs promote micronuclei generation. A,B) Representative microscopic images of micronuclei (MN) (indicated by arrowheads) in WT, *Alr^–/‐^*, and *Asc^–/–^* BMDMos following *γ*‐irradiation (9 Gy) (A) and corresponding quantification of average MNs/cell 48 h after irradiation (B) Scale bar: 10 µm. Data shown as mean ± s.e.m from 3 independent experiments representing 5 different microscopic fields. (*n* = 5) with over 100 cells. C,D) Microscopic images of MN (indicated by arrowhead) in GFP‐NLS‐ and GFP‐ALRs‐expressing HEK293 cells following *γ*‐irradiation (9 Gy) (C) and corresponding quantification of average MNs/cell 24 h after irradiation (D) Scale bar: 10 µm. D). Graphs shown as mean ± s.e.m. from 3 independent experiments representing ten different microscopic fields (*n* = 10) with over 100 cells. Data in (B,D) are presented as mean ± s.e.m. Statistical significance is assessed using one‐way ANOVA followed by Tukey's multiple comparisons test. ns: not significant, **P < 0.01, ****P < 0.0001.

Micronuclei are key hallmarks of genome destabilization and arise when broken chromosomes mis‐segregate during nuclear division.^[^ ^7^ ^]^ Following irradiation*, Alr^–/–^* BMDMos had fewer micronuclei, in contrast to WT or *Asc^–/–^* BMDMos which exhibited comparable level of micronuclei (**Figure** **3**A,B). Conversely, expression human AIM2, IFI16, IFIX, and MNDA in HEK293 cells which lack endogenous expression of ALRs resulted in enhanced micronuclei formation (Figure 3C,D). However, ablation or exogenous expression ALRs had no significant effect on cell cycle (Figure S3, Supporting Information), demonstrating that ALRs do drive genome destabilization and that this is unrelated to a possible effect on cell cycle.

### 2.4 ALRs Promote Genome Destabilization by Inhibiting DNA Repair

**Figure 4:**
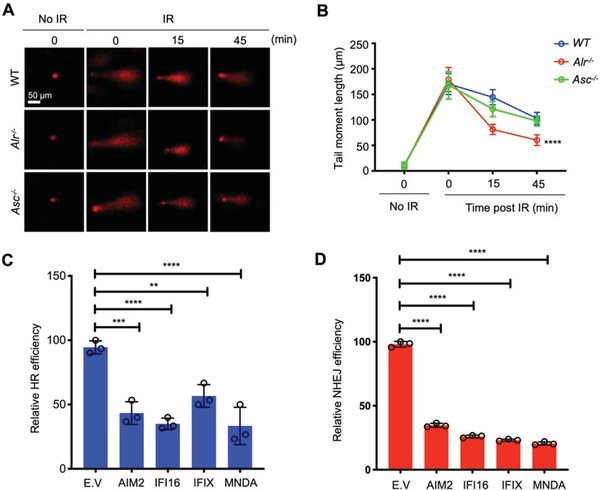
ALRs are inhibitors of double‐strand break DNA repair. A) Representative alkaline comet tails of WT, Alr‐/‐ and Asc‐/‐ BMDMos exposed to *γ*‐irradiation (9 Gy) on ice, then incubated at 37 °C to allow DNA repair for indicated duration (scale bar: 50 µm). B) Corresponding quantification of the comet tail moments from 10 different fields (*n* = 10) with over 100 comets of three independent experiments. C,D) Effect of ALRs on HR and NHEJ efficiency using GFP‐reporter assays. Data in (B) are presented as mean ± s.e.m. Statistical significance is assessed using one‐way ANOVA followed by Tukey's multiple comparisons test. Data in (C,D) are presented as mean ± sd, *n* = 3. Statistical significance is assessed using one‐way ANOVA followed by Tukey's multiple comparisons test. **P < 0.01, ****P < 0.0001. See also Figures S4 and S5, Supporting Information.

Genotoxic cell death and micronuclei formation result primarily from double‐stranded DNA breaks (DSB). Using the alkaline and neutral comet assays, next, we assessed whether ALRs do impact the resolution of DSBs. We found *Alr^–/–^* BMDMos to be more adept at resolving DSBs following irradiation. This was in contrast to *Asc^–/–^* BMDMos which repaired DSBs as efficiently as WT BMDMos (**Figure** **4**A,B and Figure S4A–D, Supporting Information). Conversely, expression of ALRs in HEK293T cells impeded DSB repair (Figure S4E,F, Supporting Information). Together, these data demonstrate that ALRs fulfill a distinct and shared function outside the innate immune system: they inhibit DNA repair, thereby promote genome destabilization, micronuclei formation, and genotoxic cell death.

Repair of DSB occurs via the homologous recombination (HR) or the non‐homologous end joining (NHEJ). To examine the DSB repair pathway impeded by ALRs, we used the GFP reporter assays for HR and NHEJ.^[^ ^8^ ^]^ All the human ALRs (AIM2, IFI16, IFIX, and MNDA) inhibited both the HR and NHEJ repair (Figure 4C,D and Figure S5, Supporting Information), indicating that these proteins do impede genome repair likely by regulating an upstream process fundamental for both DNA repair pathways.

### 2.5 ALRs Impede the Recruitment of DNA Repair Proteins to Damage Sites

**Figure 5:**
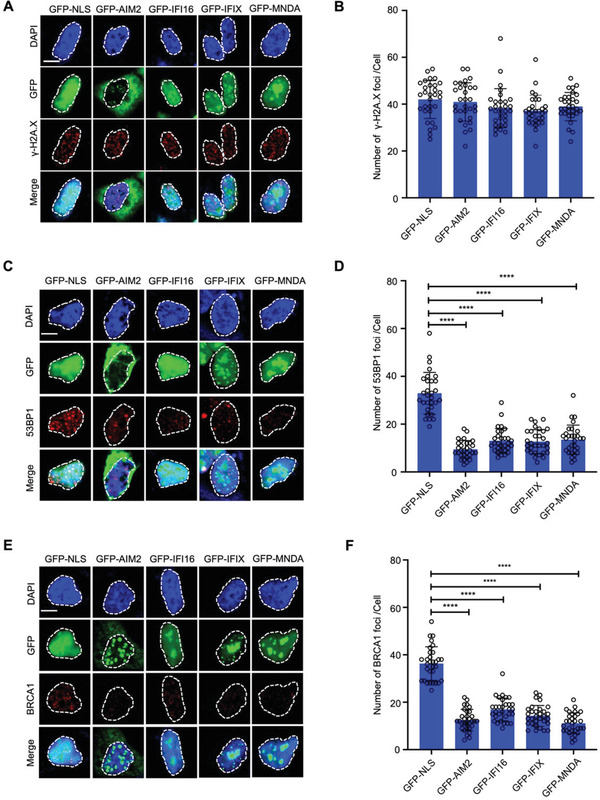
ALRs impede the recruitment of DNA repair proteins to damage sites. A–C) Immunofluorescence images of *γ*‐H2A.X (A), 53BP1 (B), and BRCA1 (C) in GFP‐NLS‐ and GFP‐ALRs‐ expressing HEK293 cells 1 h post *γ*‐irradiation (9 Gy). Scale bar = 10 µm. D–F) Corresponding quantification of *γ*‐H2A.X (D), 53BP1 (E), and BRCA1 (F) foci per nucleus. Graphs show as mean ±s.e.m, *n* = 30. Statistical significance in (D–F) is assessed using one‐way ANOVA followed by Tukey's multiple comparisons test. ****P < 0.0001. See also Figure S6, Supporting Information.

Phosphorylation of H2A.X is a key proximal signaling event enabling the recruitment of repair proteins to damage sites. Time‐course analysis of irradiated HEK293 cells transduced with ALRs revealed that ALRs (AIM2, IFI16, IFIX, and MNDA) had no effect on H2A.X phosphorylation (*γ*H2A.X) (Figure 2G,H, **Figure** **5**A,B, and Figure S6A,D, Supporting Information). However, these proteins dramatically inhibited the assembly of BRCA1 and 53BP1, key checkpoint proteins for the HR and NHEJ repair respectively^[^ ^9^ ^]^ (Figure 5C–F and Figure S6B,C,E,F, Supporting Information). This led us to conclude that ALRs are inhibitors of key repair events downstream of *γ*H2A.X but upstream of BRCA1 and 53BP1 foci formation.

### 2.6 ALRs are Nuclear Proteins

**Figure 6:**
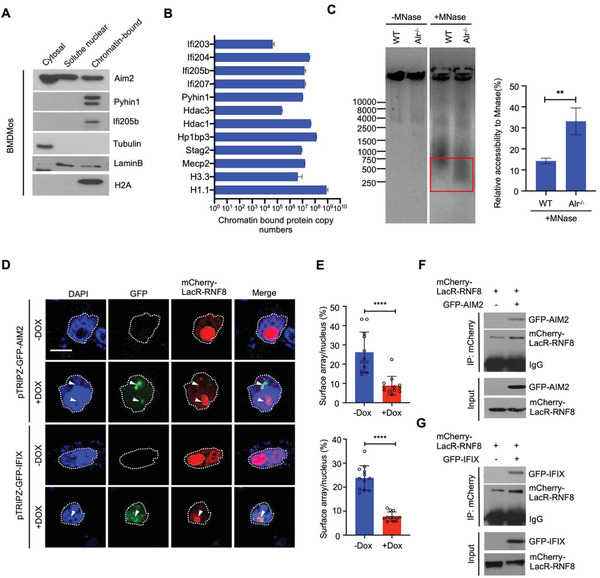
ALRs impede chromatin decompaction vital for DNA repair. A) Immunoblot analysis of ALRs in sub‐cellular fractions of BMDMos. B) The relative abundance of ALRs and other proteins on chromatin isolated from BMDMos. C) ALRs compact and hence impede chromatin accessibility. Chromatin isolated from WT or Alr‐/‐ BMDMos incubated (or not) with micrococcal nuclease (Mnase) and then analyzed by agarose gel electrophoresis. Relative accessibility of chromatin to Mnase estimated as % shift of indicated DNA from high to low molecular weight (*n* = 3). D) ALRs impede RNF8‐induced chromatin decompaction. Confocal images of mCherry‐LacR‐RNF8 and doxycycline‐induced GFP‐AIM2/IFIX (white arrowhead indicate co‐localization of GFP‐ALRs and mCherry‐LacR‐RNF8). Scale bar = 10 µm. E) Quantification of the relative array size (surface of the array/surface of the nucleus). Graphs show mean ± s.e.m. representing (*n* = 12) different microscopic fields with over 100 cells. Statistical significance is assessed using unpaired two‐tailed Student's *t*‐test. ****P < 0.0001. F,G) ALRs co‐immunoprecipitate with mCherry‐LacR‐RNF8. See also Figures S7–S9, Supporting Information.

ALRs such as AIM2 are assumed to be cytosolic. However, by subcellular fractionation and fluorescence microscopic analysis, we found that mouse and human ALRs were highly abundant in the nucleus as chromatin‐bound (**Figure** **6**A, Figures S7 and S8, Supporting Information). Further, quantitative proteomic analysis revealed that the copy number of endogenous ALRs on chromatin was comparable to many proteins known to be involved in chromatin structure, for example, histones (H1.1, H3.3), histone deacetylases (HDAC1, HDAC3), and heterochromatin protein 1 binding protein 3 (HP1BP3) (Figure 6B). While all the ALRs analyzed were found to be present in the nucleus, we also noted that AIM2 was mainly cytosolic and tended to aggregate upon irradiation. This was in contrast to IFI16, IFIX (Pyhin1), MNDA, and Ifi205 that were predominantly in the nucleus (Figure 5A,C,E, Figure 6A, Figures S7A–D and S8). The preferential nuclear accumulation of ALRs such as IFI16 or IFIX is consistent with reported presence of nuclear localization signals in these proteins.^[^ ^10^ ^]^

### 2.7 Nuclear ALRs Impede Chromatin Decompaction Vital for DNA Repair

The higher‐ordered structure of the chromatin is a significant barrier to efficient DNA repair. Hence, among the earliest events in DNA repair is chromatin decompaction to allow the recruitment of the DNA repair machinery to the damage sites.^[^ ^11^ ^]^ Given the ability of ALRs to interact with DNA and form filamentous structures through self‐oligomerization,^[^ ^12^ ^]^ we reasoned that ALRs might be promoting compaction of bound chromatin into higher‐ordered state. To directly assess the impact of endogenous ALRs on chromatin compaction, we isolated chromatin from WT and *Alr^–/–^* BMDMos and tested their accessibility to micrococcal nuclease (Mnase). Chromatin from *Alr^–/–^* BMDMos was more sensitive to Mnase digestion (Figure 6C). To verify the impact of ALRs on DNA repair‐associated chromatin decompaction, we utilized the AO3 reporter cell system for monitoring chromatin decompaction. The AO3 cells contain genomic insertions of multiple copies of the *Escherichia coli* lactose operon (LacO) sequence within a heterochromatic region.^[^ ^13^ ^]^ Upon chromatin decompaction, this chromatic region expands and this expansion can be visualized by expressing fluorescent (mCherry)‐tagged *E. coli* lactose repressor protein (LacR).

The E3 ligase RNF8, is a component of the DNA repair machinery that mediates chromatin decompaction to allow the recruitment of repair proteins including BRCA1 and 53BP1 to damage sites.^[^ ^9^ ^]^ Tethering mCherry‐tagged RNF8 to the compacted LacO chromosome region by fusing it to *E. coli* LacR causes extensive remodeling of this chromatic region into a decompacted, lacO array readily visible by fluorescence microscopy^[^ ^9^ ^]^ (see schematic overview in Figure S9A, Supporting Information). Thus, to model DNA repair‐associated chromatin remodeling and study if this is affected by ALRs, we expressed mCherry‐LacR‐RNF8 in AO3 cells. Compared to the mCherry‐LacR control, mCherry‐LacR‐RNF8 caused an expansion and transition of the LacO array into the decompacted DAPI‐weak regions (Figure S9B,C, Supporting Information). Remarkably nuclear ALRs caused a dramatic inhibition of RNF8‐mediated chromatin decompaction. Further, probably because of both their tethering to chromatin, nuclear ALRs and mCherry‐LacR‐RNF8 were partially co‐localized and co‐immunoprecipitated (Figure 6D–G). Thus, chromatin‐bound ALRs do indeed impede chromatin relaxation required for the access of DNA repair components to damage sites.

## 3 Conclusion and Discussion

The immune system and DNA repair are the primary surveillance mechanisms our bodies rely on for protection against exogenous and endogenous threats as well as for tissue repair. Hence, defects in these defense systems lie at the core of most health afflictions. Emerging evidence suggests that the immune and DNA repair systems are interdependent.^[^ ^14^ ^]^ However, the components linking these biological systems and their mechanisms remain poorly defined.

ALRs are interferon‐inducible proteins originally identified by virtue of their absence or repression in cancer cells.^[^ ^5^ ^]^ This repression is thought to promote tumorigenesis^[^ ^5^, ^15^ ^]^ and has been linked to poor patient survival.^[^ ^5e^ ^]^ Until now, much of the research on ALRs have largely focused on the immune‐modulatory function of two members: AIM2 which mediates inflammasome activation^[^ ^2b,c^ ^]^ and the IFI16 that potentiates inflammasome activation^[^ ^16^ ^]^ or the cGAS‐STING pathway for type I interferon response.^[^ ^14^, ^17^ ^]^ Here we demonstrate that the ALR family proteins have a distinct and common function outside the immune system: regulation of chromatin structure and repair. Mechanistically, we propose a model whereby, by binding to the chromatin and undergoing self‐oligomerization, nuclear ALRs do promote chromatin compaction, thus limiting the access of DNA repair machinery to damaged sites (Figure S9D, Supporting Information).

These findings have important ramification for understanding the impact of ALRs on health and disease. We show that by impeding DNA repair, nuclear ALRs promote the accumulation of DNA lesions, resulting in p53‐driven genotoxic cell death. This can be deleterious or beneficial depending on the context. For instance, whereas contributing to genotoxic tissue injury as we have shown herein, by driving the genotoxic death of cells with irreversibly damaged genomes, ALRs may help to eliminate potentially cancerous cells. Because ALRs are frequently lost in many cancer cells,^[^ ^5^ ^]^ we posit that repression of ALRs might be among the cellular adaptations favoring cancer development. The non‐canonical function of ALRs described here might account for the previously reported immune‐independent tumor‐suppressive effects of ALRs such as AIM2.^[^ ^15^ ^]^ In light of the present findings, and because the expression of ALRs varies from individual to individual,^[^ ^18^ ^]^ we also posit that differential expression of ALRs could be among the factors influencing how specific individuals or cancer cells respond to irradiation and chemotherapy.

A key principle of self‐non‐self‐discrimination by the innate immune system is based on the assumption that innate immune DNA sensors such as ALRs are exclusively in the cytosol away from genomic DNA in the nucleus. The demonstration herein that ALRs are chromatin‐bound proteins raises the question of how nuclear ALRs, for example, AIM2 are restrained to avert unwarranted activation by self‐DNA. Recently, Cyclic GMP‐AMP synthase (cGAS), another key intracellular innate immune DNA sensor was also found to be a chromatin‐bound protein that impedes DNA repair.^[^ ^14b^ ^]^ In the nucleus, cGAS is thought to be sequestered in an auto‐inhibitory state by chromatin.^[^ ^19^ ^]^ It is conceivable that similar regulatory mechanisms might account for the immune unresponsiveness of nuclear ALRs to genomic DNA. Further, the inactivity of nuclear ALRs could also in part be due to post‐translational modifications yet to be elucidated.

DNA damage‐induced inflammation is thought to result from the release of damaged self‐DNA from the nucleus and its subsequent activation of the cytosolic DNA sensors.^[^ ^14^, ^20^ ^]^ The present study reveals that by impeding genome repair, chromatin‐bound ALRs do promote micronuclei generation thus facilitating accumulation of immunogenic genomic DNA fragments in the cytosol. Thus, although presumably not directly activated by genomic DNA, by accelerating genome destabilization, chromatin‐bound ALRs could amplify DNA damage‐induced immune activation. This may for example account for the reported potentiation of DNA damage‐induced cGAS‐STING‐IFN‐I signaling by ALRs such as IFI16.^[^ ^14^, ^17^ ^]^

To conclude, DNA damage and immune activation are key determinants of the outcome of radio/chemotherapy. Our study uncovers ALRs as important molecules linking the immune and DNA repair system and highlights their potential as biomarkers for predicting patient response to radiation and chemotherapy. Further, it suggests that ALRs are possible drug targets for mitigating tissue injury caused by these treatments.

## 4 Experimental Section

### Mice

All the mice in this study were on C57BL/6 background. Alr^–/–^ and p53^–/‐^ mice were obtained from the Jackson Laboratory (stock # 029472 and #002101 respectively). Asc^–/–^ mice were obtained from Genentech, South San Francisco, USA. Alr^–/–^ mice and Asc^–/–^ mice were crossed with each other to generate the Alr^–/–^ Asc^–/–^ mice. p53^–/‐^ mice and Alr^–/–^ mice were crossed with each other to generate the p53^–/–^Alr^–/–^ mice. All mice were bred in specific pathogen‐free animal facility of Umeå center for comparative Biology and experiments were carried out according to the guidelines set out by the Umeå Regional Animal Ethic Committee (Umeå Regionala Djurförsöksetiska Nämnd), Approval no. A25‐19.

### BM Depletion

For irradiation, mice were placed in a Gammacell 40 irradiator (MDS Nordion) with a 137Cs gamma‐ray source. Radiation was given as a single dose of 1 Gy per min for 9 min (total dose of 9 Gy). 10 h after irradiation mice were sacrificed and BM cells isolated, counted, and analyzed by flow cytometry for the following cell populations, hematopoietic progenitors (c‐Kit^+^Sca‐1^+^), monocytes (Ly6C^+^) and neutrophils (Gr1^+^) (see gating strategy in Figure S10, Supporting Information). The total BM cells or specified cell populations in the femur were calculated and expressed as relative (percentage) to non‐irradiated controls.

### Antibodies and Reagents

Pam3CSK4 and poly(dA:dT) were purchased from Invivogen. DAPI, Antibodies against *β*‐Actin and *β*‐Tubulin were purchased from Sigma‐Aldrich. Caspase‐1 p20 antibody (Clone 4B4.2.1) was obtained from Genentech, San Francisco USA. IL‐1*β* antibody was from R&D Systems. Antibodies against H2A, H2A.X, *γ*‐H2A.X, P53, and p‐P53(S15) were from Cell Signaling Technology. 53BP1 antibody was obtained from Novus biologicals. Anti‐HA, anti‐GFP, and anti‐BRCA1 antibodies were purchased from Santa Cruz Biotechnology. Pyhin1 and Ifi205b(Mnda) antibody were purchased from Mybiosource. mCherry, Alexa488‐Anti‐Sca‐1 was from Invitrogen and PE/Cy7‐Anti‐cKit, V450‐Anti‐Ly6C, FITC‐Anti‐GR1 were from BD Pharmingen.

### Plasmid and Construct Cloning

The coding sequence of human AIM2, IFI16, IFIX, and MNDA were cloned into pcDNA3.1+ vector to generate pcDNA‐AIM2, pcDNA‐IFI16, pcDNA‐IFIX, pcDNA‐MNDA. pTRIP‐SFFV‐EGFP‐NLS(GFP‐NLS) was from Addgene (plasmid #86677). GFP‐AIM2, GFP‐IFI16, GFP‐IFIX, and GFP‐MNDA were cloned into pTRIP vector to generate pTRIP‐GFP‐AIM2, pTRIP‐GFP‐IFI16, pTRIP‐GFP‐IFIX, pTRIP‐GFP‐MNDA. For inducible expression of ALRs, GFP‐AIM2, GFP‐IFI16, GFP‐IFIX, and GFP‐MNDA were cloned into pTRIPZ vector to generate pTRIPZ‐GFP‐AIM2, pTRIPZ‐GFP‐IFI16, pTRIPZ‐GFP‐IFIX, pTRIPZ‐GFP‐MNDA. The mCherry‐LacR and mCherry‐LacR‐RNF8 plasmids^[^ ^9^ ^]^ were a gift from Nico Dantuma lab, the Karolinska Institute, Stockholm.

### Cells and Cell Culture

HEK239T, HEK293, and HCT116 cells were cultured under 5% CO_2_ at 37 °C in Dulbecco's modified Eagle medium (DMEM, high glucose, GlutaMAX) (Life Technologies) containing 10% (v/v) fetal calf serum (FCS, GIBCO), 1% (v/v) penicillin (100 IU mL^−1^) +streptomycin (100 µg mL^−1^). Bone‐marrow‐differentiating monocytes (BMDMos) were generated by culturing the mouse BM cells in IMDM medium (GIBCO, Life Technologies) supplemented with 10% (v/v) FCS (GIBCO, Life Technologies), 1% (v/v) penicillin (100 IU mL^−1^)/streptomycin (100 µg mL^−1^), 2 mM glutamine (Sigma‐Aldrich) and 10% (v/v) L929 conditional medium and maintained in 5% CO_2_ at 37 °C. Cells were used for experiment 4 days after start of differentiation. AO3 hamster cells, containing a 90‐Mbp amplification of LacO sequences and flanking DNA^[^ ^9^, ^13^ ^]^ were cultured in a 1:1 mixture of DME/Ham's F12 medium supplemented with antibiotics and 20% FCS.

### Generation of Stable Overexpression Cell Lines

HEK293T cells were transfected with psPAX2, pMD2.G plasmids, and the lentiviral vector pTRIP or inducible lentiviral vector pTRIPZ containing an open reading frame of GFP‐NLS or GFP‐ALRs by using Lipofectamine LTX. Supernatants containing lentiviral particles were harvested at 48 h. HEK293, HCT116, and AO3 cells were then transduced with the lentiviral vectors by directly adding supernatant together with polybrene (5 µg mL^−1^) to cells. 2 days later, GFP‐positive cells were sorted by flow cytometry and propagated further. Transduced cells were selected by puromycin (2 µg mL^−1^) for at least one week before use for experiments.

### Cell Death Assays

For irradiation‐induced cell death, BMDMos synchronized at G2/M by incubating with 100 nM nocodazole for 12 h were *γ*‐irradiated then release and cell viability determined at indicated time points by XTT assay (Sigma‐Aldrich) according to the manufacturer's instructions. Absorbance was measured with a spectrophotometer (Tecan Infinite M200 Microplate Reader) at 450 nm with a reference wavelength at 650 nm. Relative number of dead cells as compared to the number of cells without treatment was expressed as percent cell death using the following formula: cell death (%) = 100% − 100% X(A450 of treated cells/A450 of untreated cells).

### LDH Release Assay

BMDMos primed (or not) with Pam3CSK4 (500 ng mL^−1^) for 4 h were transfected with 1 µg mL^−1^ poly(dA:dT) and analyzed for the release of Lactate Dehydrogenase (LDH) using a kit (ThermoFisher, Cat. No. C20300) according to the manufacturer's instructions.

### Cell Cycle Analysis

Following the individual treatments (i.e., nocodazole treatment, or irradiation), cells were washed twice in PBS, then fixed in cold 70% ethanol for 30 min at 4 °C. Thereafter, they were washed and treated with RNase to remove RNA. After washing, cells were stained with DAPI at 4 °C. Flow cytometry was performed on BD LSR II flow cytometer, and the data were analyzed with FlowJo software.

### Immunofluorescence

Cells were seeded and cultured on glass coverslips in 12‐well plate and fixed in 4% paraformaldehyde in PBS for 20 min at room temperature. Cells were permeabilized in 0.5% Triton X‐100 for 10 min. Slides were blocked in 5% normal goat serum (NGS) and incubated with primary antibodies diluted in 1% NGS overnight at 4 °C. Samples were then incubated with indicated secondary antibodies diluted in 1% NGS at RT for 1 h, before staining with DAPI for 15 min at room temperature. Coverslips were mounted using Dako Fluorescence Mounting Medium (Agilent) and imaged using Nikon confocal microscope (Eclipse C1 Plus). All scoring was performed under blinded conditions. *γ*‐H2AX, BRCA1, and 53BP1 foci were counted from 30 microscopic fields containing approx. 300 cells from 3 independent experiments per time point.

### HR and NHEJ Reporter Assays

Homologous recombination (HR) and NHEJ repair in HEK293T cells were measured as described previously using the DR‐GFP stable cells^[^ ^8b^ ^]^ and EJ5‐GFP stable cells.^[^ ^8a^ ^]^ Briefly, 0.5 × 10^6^ HEK293T stable reporter cells were seeded in 6‐well plates co‐transfected with 2 µg I‐SceI expression plasmid (pCBASce) and either 4 µg pcDNA‐ALRs or empty pcDNA vector. 48 h post‐transfection, cells were harvested and analyzed by flow cytometry analysis for GFP expression. Means were obtained from three independent experiments.

### Subcellular Fractionation and Immunoblotting

To isolate the cytoplasmic, soluble nuclear fraction and chromatin‐bound fraction, the Subcellular Protein Fractionation Kit (Thermo Fisher) was applied according to the manufacturer's instructions. For other assays, cells grown in culture were trypsinized, pelleted, washed, and resuspended in a mild Nonidet P‐40 lysis buffer (1% NP‐40, 50 mM Tris–HCl, 150 mM NaCl, pH 7.5, 1 mM NaF, 2 mM PMSF, protease inhibitor cocktail [Roche Applied Science], 1 mM sodium orthovanadate, and 10 mM sodium pyrophosphate). Lysates were centrifuged at 10 000 g for 15 min and proteins in supernatants were quantified by BCA reagent (Thermo Fisher Scientific). Proteins were resolved in SDS–PAGE, transferred to nitrocellulose membrane (Amersham, 0.45 µm NC), and immunoblotted with specific primary antibodies followed by HRP‐conjugated secondary antibodies. Protein bands were detected by SuperSignal West Pico or Femto Chemiluminescence Kit (Thermo Fisher Scientific).

### Inflammasome Activation Analysis

BMDMos seeded in the density of 1.5 × 10^6^ cells well^−1^ were primed (or not) with 500 ng mL^−1^ Pam3CSK4 for 4 h and then irradiated with indicated irradiation dose or transfected with 1 µg mL^−1^ poly(dA:dT) using Lipofectamine 2000. Supernatants were collected. Proteins were precipitated using chloroform:methanol extraction and resuspended in 2× Laemmli buffer. Cells were lysed in 2× Laemmli buffer. Samples were separated on 13.5% SDS‐PAGE gel and analyzed for activation of Caspase‐1 and IL‐1*β* by immunoblotting, as described in the section above.

### Comet Assay

Cells were *γ*‐irradiated in a 137Cs gamma‐ray source (Gammacell 40 irradiator, MDS Nordion) with indicated dose or treated with indicated drugs. Chromosome fragmentation was then determined by comet assay. Briefly, during irradiation all the cells were kept on ice to stop the DNA repair process then transferred to 37 °C to allow DNA repair and harvested at indicated time points for analysis. 1 × 10^5^ cells mL^−1^ in cold PBS were resuspended in 1% low‐melting agarose at 40 °C at a ratio of 1:3 vol/vol and pipetted onto a CometSlide. For alkaline comet assay, slides were immersed in prechilled alkaline lysis buffer (1.2 M NaCl, 100 mM EDTA, 0.1% sodium lauryl sarcosinate, 0.26 M NaOH PH > 13) for overnight (18–20 h) lysis at 4 °C in the dark. Slides were then carefully removed and submerged in room temperature alkaline rinse buffer (0.03 M NaOH and 2 mM EDTA, pH > 12) for 20 min in the dark. This washing step was done 2 times. Slides were transferred to a horizontal electrophoresis chamber containing alkaline rinse buffer and separated for 25 min at voltage (0.6 V cm^−1^).

For neutral comet assay, slides were immersed in prechilled neutral lysis buffer (2% sarkosyl, 0.5 M Na2EDTA, 0.5 mg mL^−1^ proteinase K, PH8.0), then moved to an incubator at 37 °C overnight (18–20 h) in the dark. After overnight lysis, slides were removed and submerged in neutral rinse buffer (90 mM Tris buffer, 90 mM boric acid, and 2 mM Na2 EDTA, pH8.5) for 20 min at room temperature. After washing twice, slides were transferred to a horizontal electrophoresis chamber containing neutral rinse buffer and separated for 60 min at voltage (0.6 V cm^−1^). Finally, slides were washed with distilled water and stained with 10 µg mL^−1^ propidium iodide and analyzed by fluorescence microscopy. 10 fields with about 100 cells in each sample were evaluated and quantified by the Fiji software to determine the tail length (tail moment length).

### Determination of Micronuclei

HEK293‐ALRs cells were exposed (or not) to *γ*‐irradiation and cultured for 24 h. BMDMos were first synchronized at G2/M by incubating with nocodazole before exposure to *γ*‐irradiation, then release and further cultured for 48 h. Cells were fixed, permeabilized in 0.5% Triton X‐100, stained with the DNA dye DAPI, then analyzed by microscopy for the presence of micronuclei. Micronuclei were defined as discrete DNA aggregates separated from the primary nucleus in cells where interphase primary nuclear morphology was normal. Cells with an apoptotic or necrotic appearance were excluded.

### Quantitative Proteomics

To estimate the copy number of proteins on chromatin, chromatin fractions were isolated from BMDMos using the subcellular Protein Fractionation Kit (Thermo Fisher) then analyzed by mass spectrometry and “protein ruler” as previously described.^[^ ^21^ ^]^

### The Micrococcal Nuclease (Mnase) Sensitivity Assay

Briefly, cells were collected and lysed in ice‐cold Nonidet P‐40 cell lysis buffer (10 mM Tris‐HCl pH 7.5, 10 mM NaCl, 3 mM MgCl_2_, and 0.4% Nonidet P‐40) with protease inhibitors and incubated on ice for 5 min, then lysate was centrifugated at 2000 × g for 5 min at 4 °C. The lysate pellet was collected and washed with lysis buffer twice, the pellet was then resuspended in 50 mL glycerol buffer (10 mM Tris‐HCl pH 7.4, 0.1 mM EDTA, 5 mM MgAc_2_, and 25% (vol/vol) glycerol), mixed with equal volume of MNase digestion buffer (50 mM KCl, 8 mM MgCl_2_, 2 mM CaCl_2_, and 100 mM Tris‐HCl pH 7.4), and incubated at 37 °C for 5 min with 1 U MNase (Thermo Fisher Scientific) per 100 mL of total reaction volume. The reactions were quenched by adding EDTA at the final concentration of 10 mM. The reaction products were separated by electrophoresis in 1% agarose gel.

### Kaplan–Meier Analysis of Gastric Cancer Patients

Kaplan–Meier curves were generated using publicly available microarray datasets of human gastric cancer patients (kmplot.com/analysis). Patients were divided according to the expression values of target genes, with expression values in the top one‐third (≈33%) range grouped as high expressers and those in the bottom one‐third range grouped as low expressers. The Affymetrix IDs for AIM2 is 206 513, IFI16 is 206 332_s, IFIX (also known as PHYN1) is 240 413, MNDA is 204 959.

### Statistical Analysis

Statistical analysis was performed by GraphPad Prism 5.0 software. All of the data shown in the histograms were the results of at least three independent experiments and are presented as the mean ± s.e.m or mean ± sd. The sample size (n) for each statistical analysis and statistical methods used to assess significant differences are indicated in figure legends. Differences between values were considered statistically significant when ^∗^ *P* < 0.05, ^∗∗^ *P* < 0.01, ^∗∗∗^ *P* < 0.001, and ^∗∗∗∗^ *P* < 0.0001.

## Conflict of Interest

The authors declare no conflict of interest.

## Author Contributions

N.O.G. conceived and supervised the study. H.J. conceived the study and performed most of the experiments. P.S. performed the in vitro inflammasome assays. N.O.G. and H.J. performed in vivo experiments and flow cytometry. N.O.G. and H.J. wrote the paper which P.S. commented on.

## Supporting information

Supporting Information

## Data Availability

Data sharing is not applicable to this article as no new data were created or analyzed in this study.
