# Supplementary material for: Nuclear AIM2‐Like Receptors Drive Genotoxic Tissue Injury by Inhibiting DNA Repair
Source: Adv Sci (Weinh). 2021 Oct 18;8(22):2102534. doi: 10.1002/advs.202102534 (PMC8596118; doi:10.1002/advs.202102534)
Supplement: Supplementary file 1 — Supporting Information [file ADVS-8-2102534-s001.pdf]

WILEY-VCH

© 2021 Wiley-VCH GmbH

ADVANCED SCIENCE
Open Access

Supporting Information

for *Adv. Sci.*, DOI: 10.1002/advs.202102534

Nuclear AIM2-like receptors drive genotoxic tissue injury by inhibiting DNA repair

*Hui Jiang, Patrycja Swacha and Nelson O. Gekara**

Supporting Information

Nuclear AIM2-like receptors drive genotoxic tissue injury by inhibiting DNA repair

*Hui Jiang, Patrycja Swacha and Nelson O. Gekara**

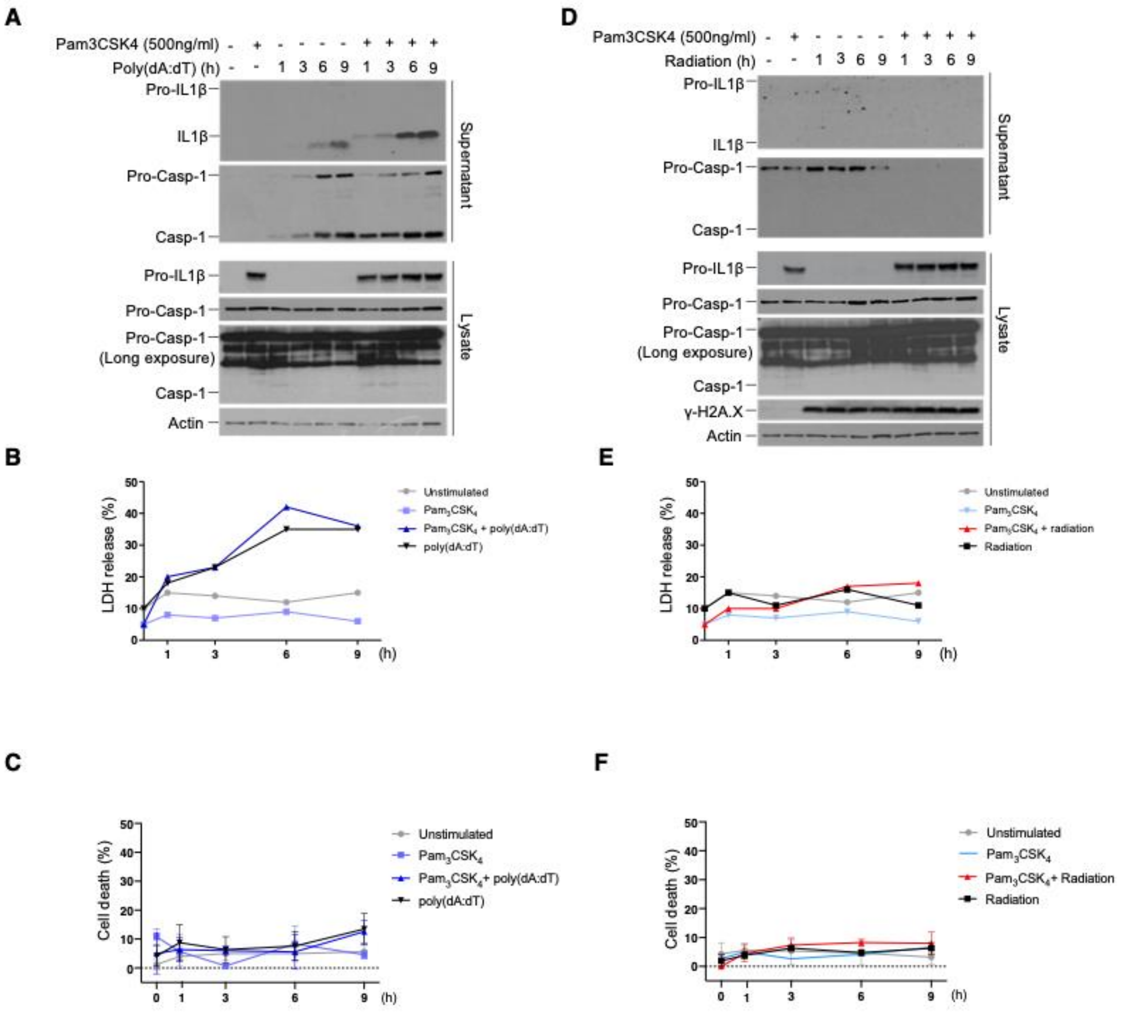

Figure S1. γ-irradiation is a poor inflammasome activator. (A) Immunoblots of Caspase-1 and IL-1β processing in poly(dA:dT) transfected BMDMos. (B) LDH release in poly(dA:dT) transfected BMDMos. (C) Cell death (XTT assay) in poly(dA:dT) transfected WT BMDMos. (D) Immunoblots of Caspase-1 and IL-1β processing in γ-irradiated (20 Gy) BMDMos. (E) LDH release in γ-irradiated (20 Gy) BMDMos. (F) Cell death (XTT assay) in γ-irradiated (20 Gy) BMDMos. **Related to Figure 1.**

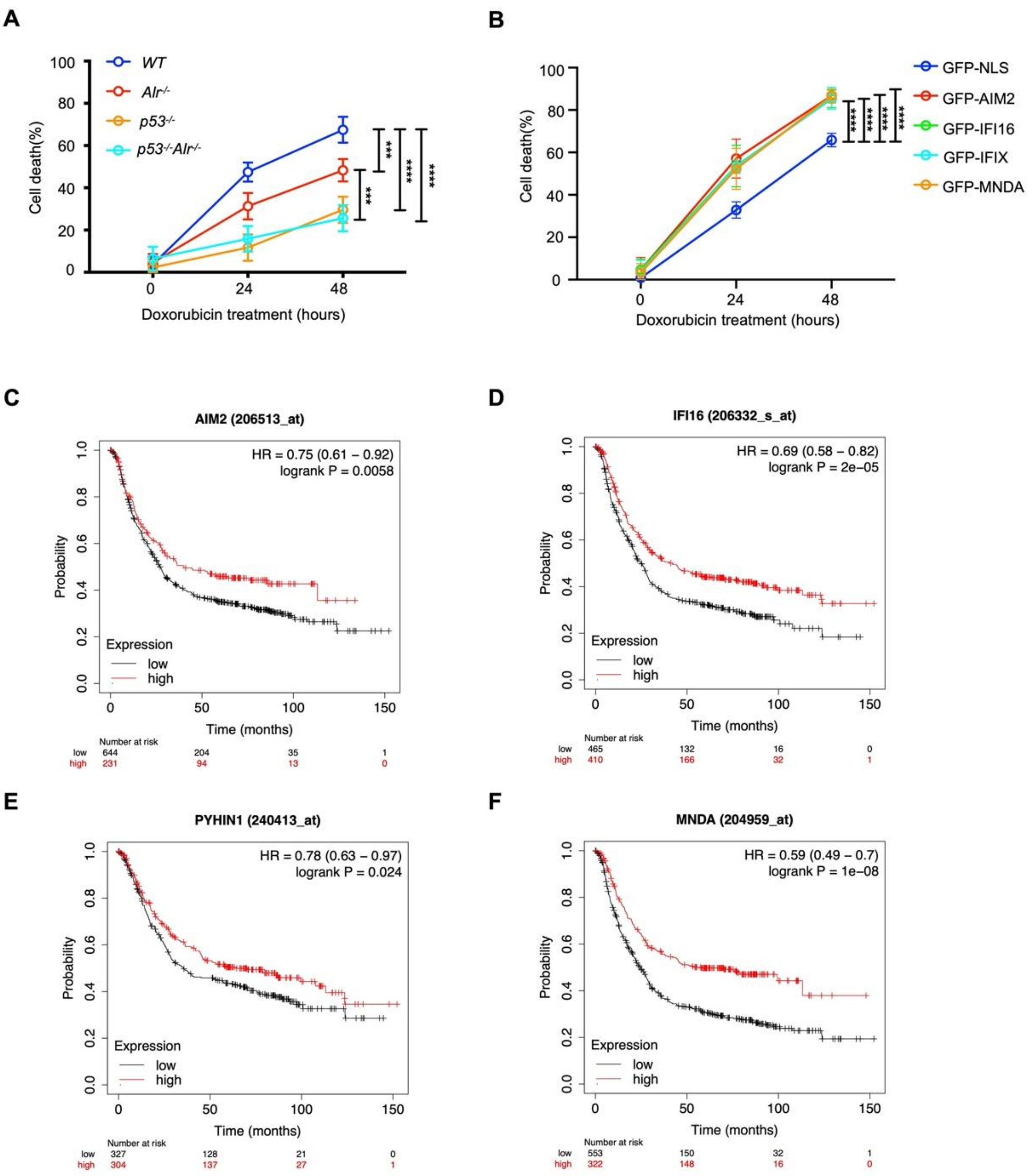

Figure S2. ALRs promote cell death and anti-cancer effects of chemotherapy. (A) Cell death (XTT assay) in WT, Alr$^{-/-}$, p53$^{-/-}$ and p53$^{-/-}$Alr$^{-/-}$ BMDMos treated with doxorubicin (2 uM) for indicated duration (n = 3, biological repeat). (B) Doxorubicin (4 uM) - induced cell death in HCT116 cells transduced with GFP-AIM2, GFP-IFI16 GFP-IFIX, GFP-MNDA or control GFP-NLS (nuclear localization signal). Data in (A, B) are presented as mean ± sd, n=3. Statistical significance in were assessed using two-way ANOVA test. ns: not significant, **P < 0.01, ***P < 0.001, ****P < 0.0001. Expression levels of ALRs positively correlate with better survival of human gastric cancer patients following chemotherapy. Kaplan–Meier curves were generated from the public microarray databases of human patients using online software (kmplot.com/analysis/). Groups of patients with high (top one-third) expression levels of the indicated genes AIM2 (C), IFI16 (D), IFIX/PYHIN1 (E), and MNDA (F), were compared with those with low expression levels (bottom one-third). The Affymetrix microarray ID for each gene is shown in in the Figure panels. Statistical significance was determined using the log-rank test. HR, hazard ratio.

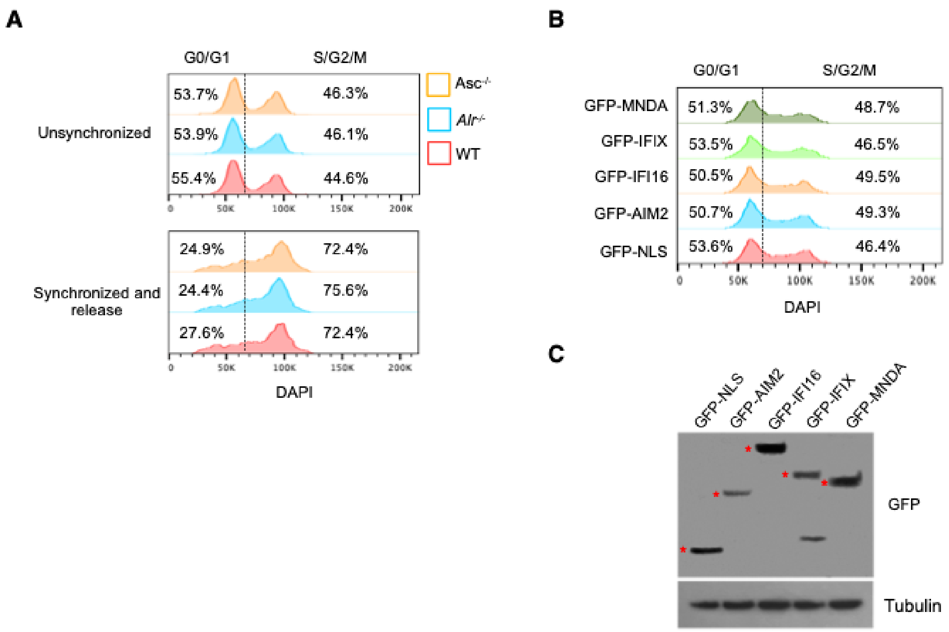

Figure S3. Effect of ALRs on cell cycle. (A) WT, *Alr*$^{-/-}$, and *Asc*$^{-/-}$ BMDMos were treated (or not) with nocodazole for 12 hours, then washed and cell cycle phase was determined by flow cytometry. (B and C) Cell cycle analysis of unsychronized HEK293 stably expressing GFP-NLS or GFP-ALRs cells (B) and corresponding immunoblots analysis of GFP-NLS and GFP-ALRs (red star indicate individual GFP-ALRs).

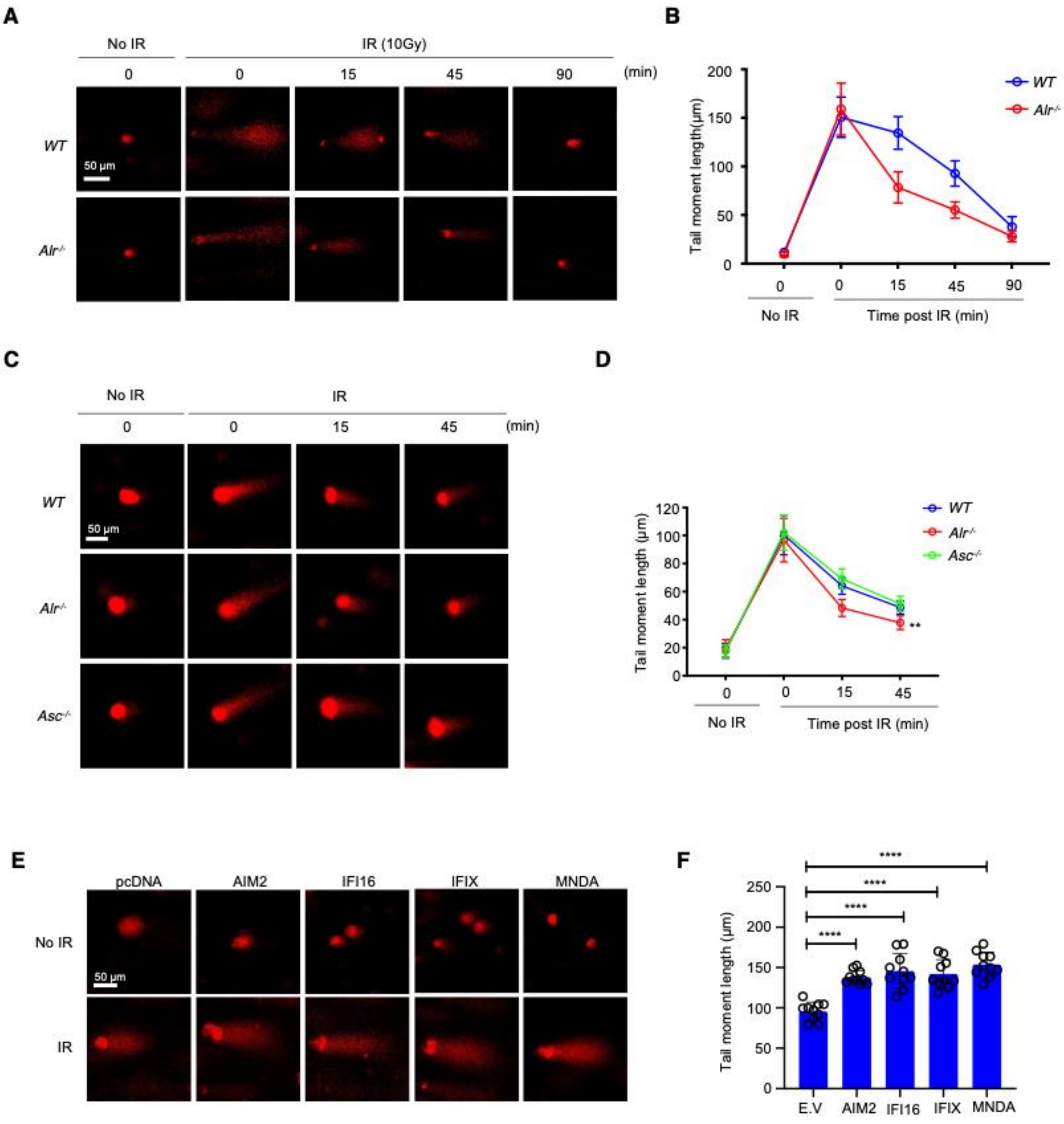

Figure S4. ALRs inhibit DNA repair. (A) Representative alkaline comet tails of WT and *Alr*$^{-/-}$ BMDMos exposed to γ-irradiation (9 Gy) on ice, then incubated at 37°C to allow DNA repair for indicated duration, sacle bar: 50 μm. (B) Corresponding quantification of the comet tail moments from 10 different fields (n=10) with over 100 comets of three independent experiments. (C) Representative neutral comet tails of WT, *Alr*$^{-/-}$ and *Asc*$^{-/-}$ BMDMos exposed to γ-irradiation (20 Gy) on ice, then incubated at 37°C for indicated duration. scale bar: 50 μm. (D) Corresponding quantification of the comet tail moments from 10 different fields (n=10) with over 100 comets of three independent experiments. (E) Alkaline comet tails 15 min after 9 Gy γ-irradiation of HEK293T transfected with empty pcDNA vector (E.V) or indicated ALRs. (F) Corresponding quantification of comet tails from 10 different fields (n=10) with over 100 comets of three independent experiments. Data are presented as mean ± s.e.m. Statistical significance in (B) was assessed using two-way ANOVA test. Statistical significance in (D) was assessed using One-way ANOVA followed by Tukey's multiple comparisons test. ****$P < 0.0001$. **Related to Figure 4.**

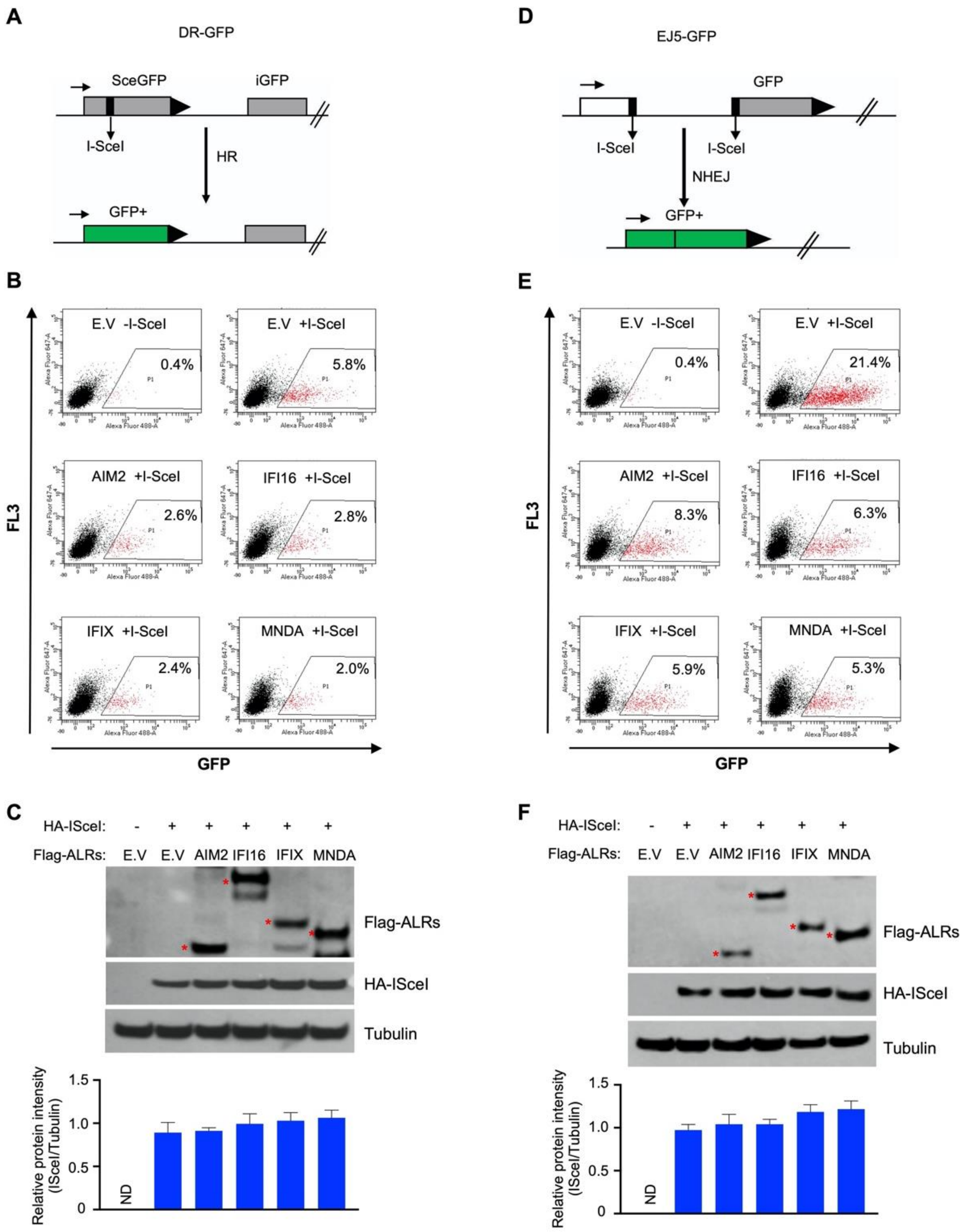

Figure S5. ALRs inhibit both HR- and NHEJ- DNA repair. (A) Schematics of HR-GFP reporter assays. (B) Representative flow cytometry data of HR-GFP reporter assay and (C) corresponding immunoblots of Flag-ALRs and HA-ISceI (and quantification of ISceI relative to tubulin n=3) in the HR-GFP reporter cells (red star indicate individual Flag-ALRs). (D) Schematics of NHEJ-GFP reporter assays. (E) Representative flow cytometry data of NHEJ-reporter assay and (F) corresponding immunoblots of Flag-ALRs and HA-ISceI (and quantification of ISceI relative to tubulin n=3) in NHEJ-GFP reporter cells (red star indicate individual Flag-ALRs). **Related to Figure 4.**

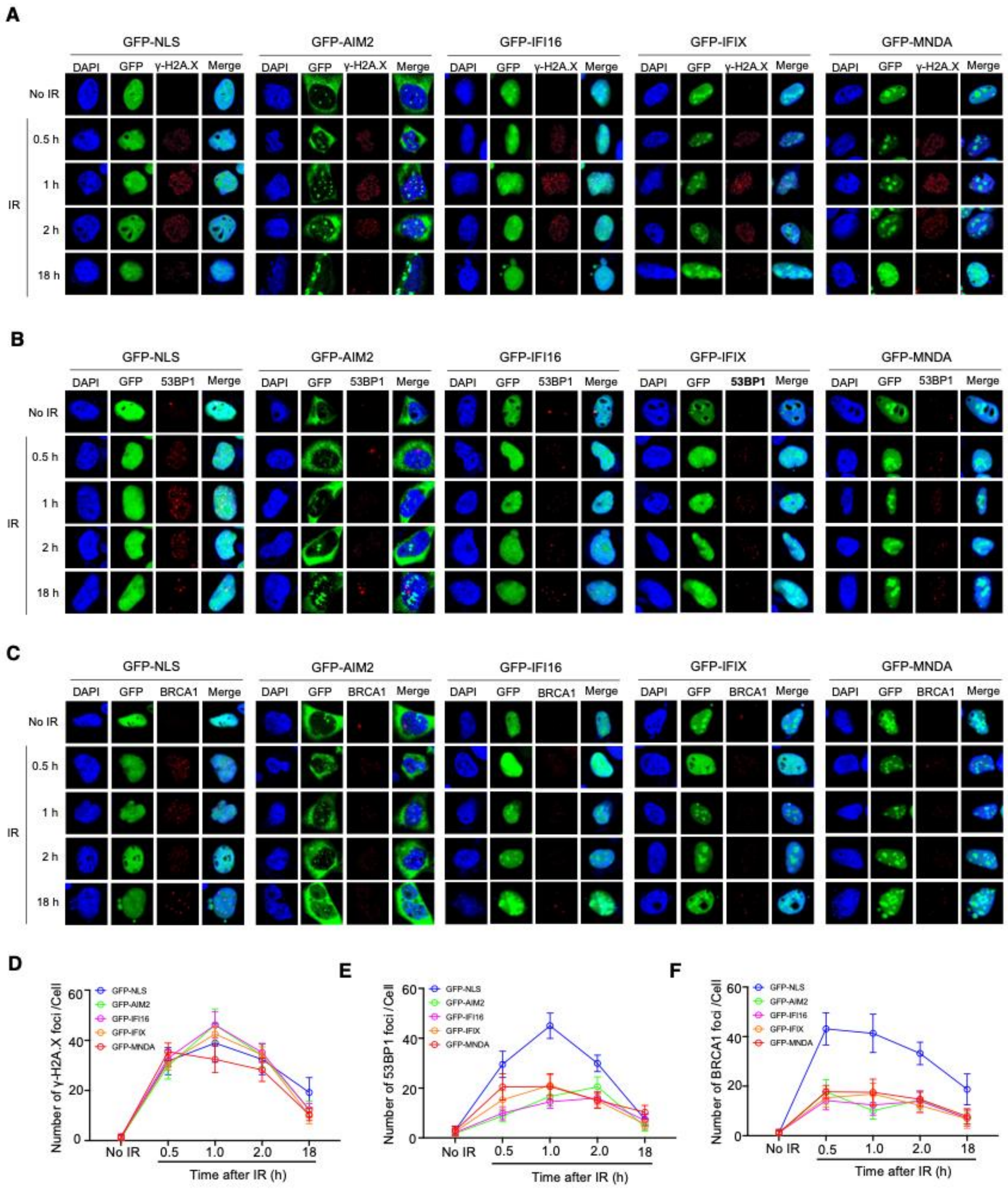

Figure S6. ALRs impede the recruitment of DNA repair proteins to damage sites. (A-C) Immunofluorescence images of γ-H2A.X (A) 53BP1 (B) and BRCA1 (C) in GFP-NLS- or GFP-ALRs-expressing HEK293 cells at indicated time points following γ-irradiation (IR; 9 Gy). Scale bar = 10 μm. (D – F) Corresponding quantification of γ-H2A.X (D) 53BP1 (E) and BRCA1 (F) foci per nucleus. Graphs show mean ± s.e.m, n = 30. Statistical significance was assessed using One-way ANOVA followed by Tukey's multiple comparisons test. **Related to Figure 5.**

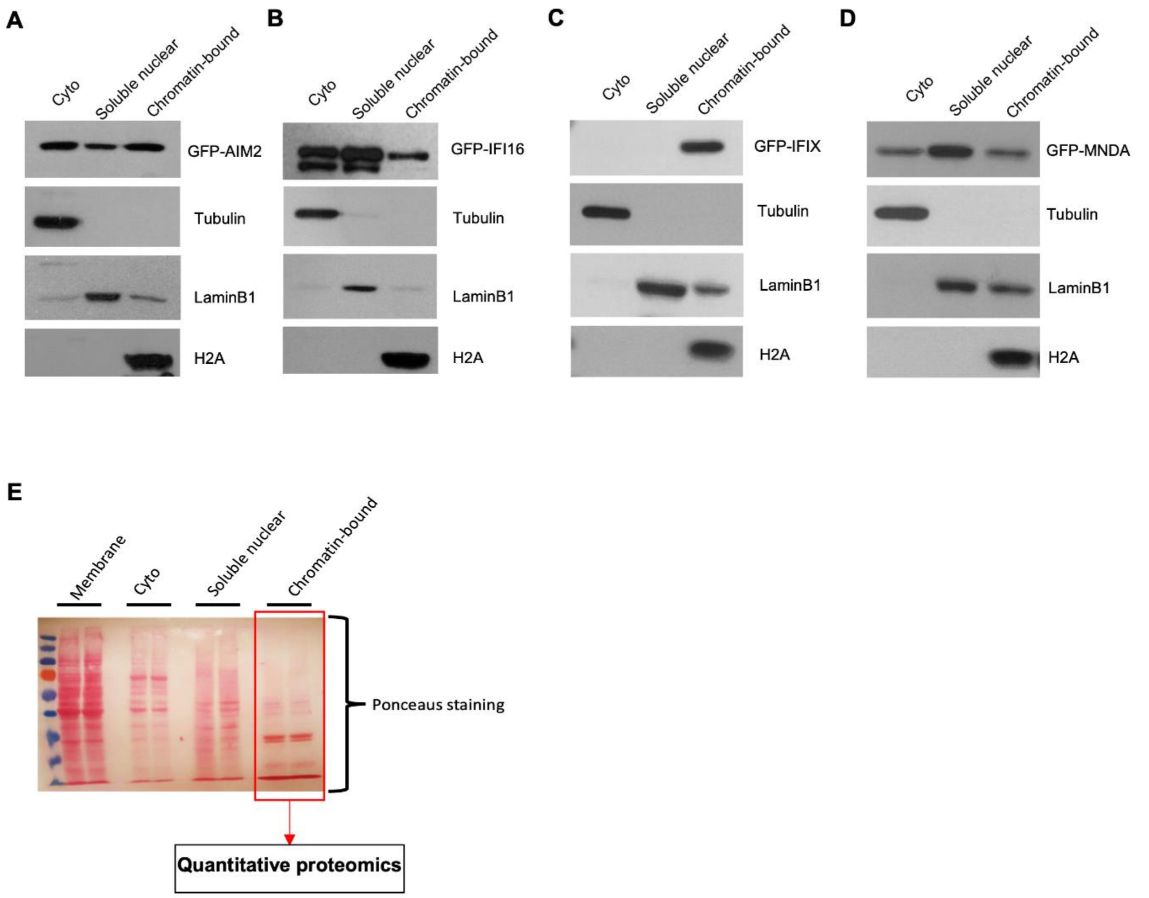

Figure S7. Subcellular distribution of ALRs. (A-D) Immunoblots of cytosolic (cyto), soluble nuclear and chromatin fractions from HEK293 cells expressing indicated GFP-ALRs. (E) Ponceau staining of BMDMos subcellular fraction used for quantitative proteomics analysis in Figure 6B. **Related to Figure 6.**

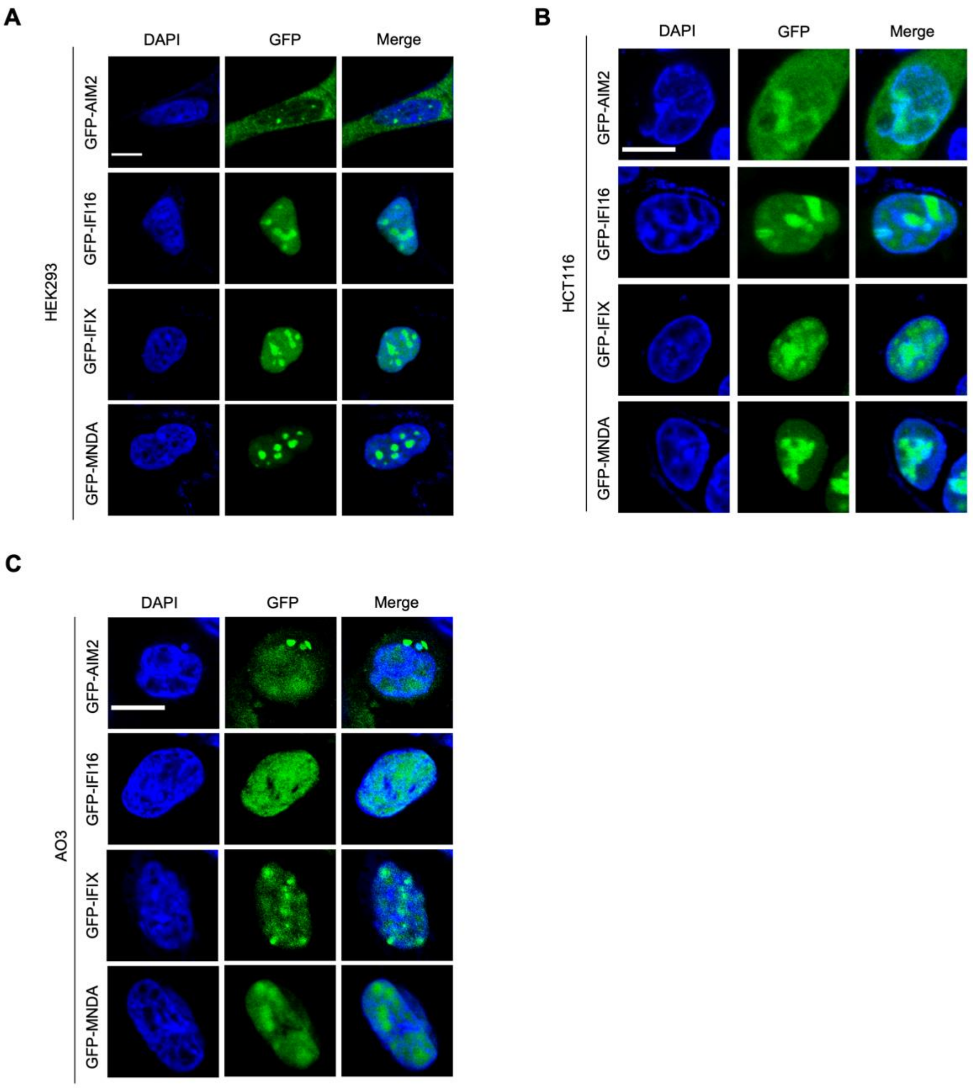

Figure S8. ALRs are abundant in the nucleus. (A-C) Microscopy analysis of indicated GFP-ALRs in HEK293 cells (A), HCT116 cells (B) and AO3 cells (C). Scale bars = 10 μm. **Related to Figure 6.**

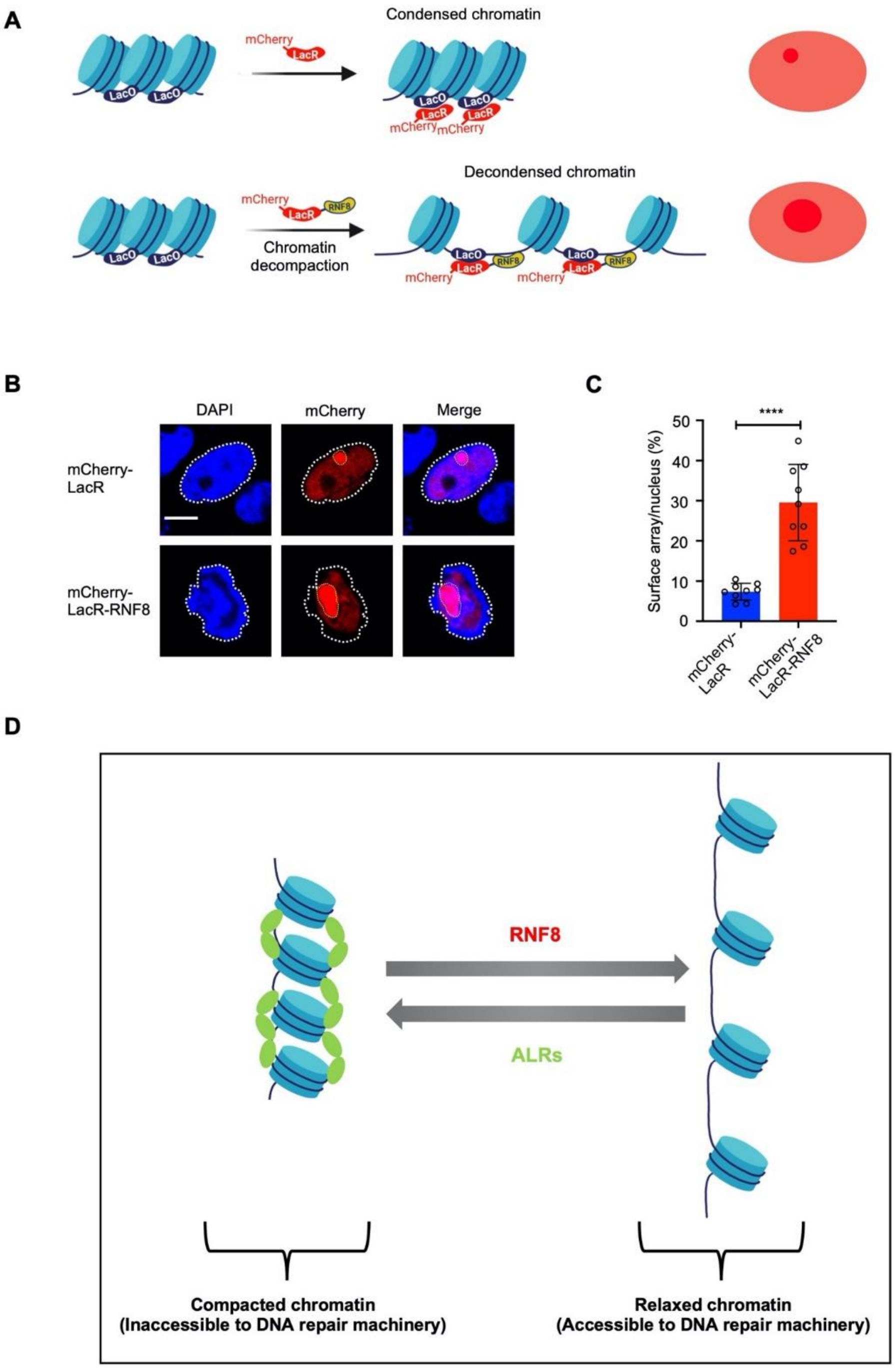

Figure S9. RNF8 drives chromatin relaxation. (A) Schematic representation of the chromatin compaction assay. (B) AO3 cells containing a 90-Mbp heterochromatic array were transfected with mCherry-LacR or mCherry-LacR-RNF8. Confocal images show the nuclear distribution of the mCherry-LacR fusion proteins. Scale bar = 10 µm. (C) Quantification of the relative array size (surface of the array/surface of the nucleus). Graphs show mean ± s.e.m. from 9 different microscopic fields each with over 100 cells. (D) Proposed model for ALR-mediated inhibition of chromatin relaxation during DNA repair. Statistical significance in (C) was assessed using unpaired two-tailed Student's t-test. ****$P < 0.0001$. **Related to Figure 6.**

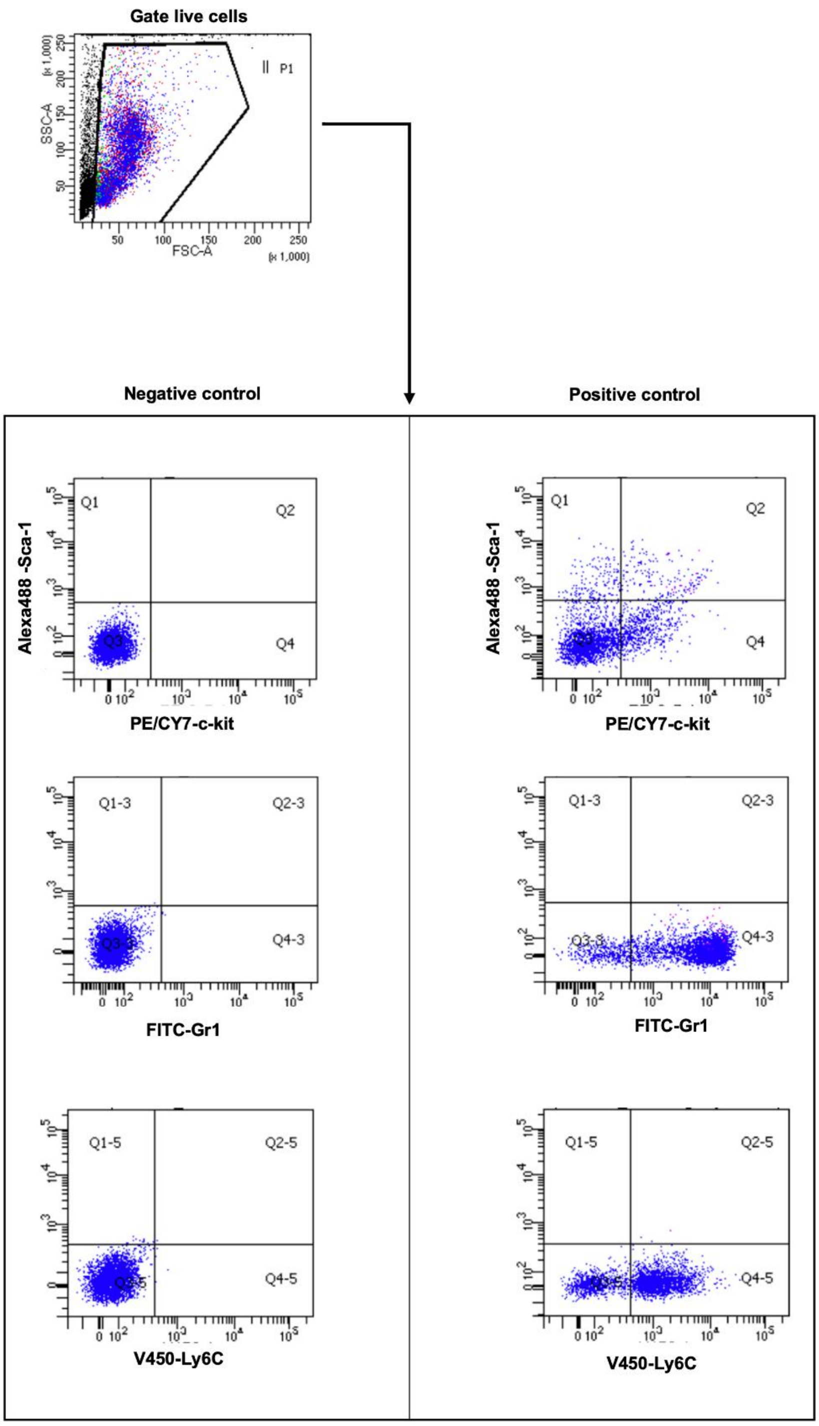

Figure S10. Flow cytometry gating strategies for analyzing bone marrow cell populations.
